# Unhealthy lifestyle profile as a risk factor for poor quality of life and mental health in chronic kidney disease patients: a comparative case–control study

**DOI:** 10.3389/fmed.2025.1547321

**Published:** 2025-04-25

**Authors:** Maryam Beyrami, Sohrab Amiri

**Affiliations:** ^1^Science and Research Branch, Department of General Psychology, Faculty of Literature, Humanities and Social Sciences, Islamic Azad University, Tehran, Iran; ^2^Spiritual Health Research Centre, Life Style Institute, Baqiyatallah University of Medical Sciences, Tehran, Iran

**Keywords:** lifestyle, chronic kidney disease, health-related quality of life, mental health, case–control

## Abstract

**Background:**

This study examined the mental health status and lifestyle of chronic kidney disease patients in comparison to a health control group. It also evaluated lifestyle factors as potential risk factors for kidney disease.

**Methods:**

The case–control comparative study included chronic kidney disease (CKD) patients aged ≥18 years and a healthy control group. The primary outcomes were lifestyle profile, health-related quality of life, psychiatric morbidity, and somatic symptoms experiences. Associations between sociodemographic characteristics, health behaviors, and the risk of CKD were investigated using logistic regression, with odds ratios (ORs) and 95% confidence intervals (CIs) calculated. Independent *t*-tests were used to compare kidney patients with the healthy control group.

**Results:**

The CKD group scored lower in most aspects of lifestyle and health-related quality of life than the healthy control group. Additionally, CKD patients exhibited poorer mental health status than the healthy control group. Factors associated with chronic kidney disease risk include female gender, history of disease, and being retired. A health-promoting lifestyle among chronic kidney disease patients had a direct relationship with high health-related quality of life. Furthermore, a health-promoting lifestyle was negatively associated with mental health disorders and somatic symptoms experiences.

**Conclusion:**

Compared to the healthy control group, CKD patients in this study reported more pain, physical complaints, and depression. A healthy lifestyle can be effective in the prevention and treatment of many physical and mental health problems. Compared to other treatments used for mental disorders, such as drug therapy or psychological therapy, lifestyle interventions can have long-lasting effects and tend to be more cost-effective for individuals.

## Introduction

Kidney disease is one of the most significant health problems affecting approximately 10% of the population (1). Awareness of this problem has been limited and is still incomplete (2). The multidimensional burden of kidney disease is increasing day by day, including prevalence, morbidity, mortality, and costs, and the disease burden is more common in low-income countries (1). According to a published report, 850 million people worldwide suffer from kidney disease, with the majority living in low-and middle-income countries. This population often has limited access to healthcare, including diagnosis, prevention, and treatment (3). Early diagnosis of kidney disease is vital to track its deterioration, progression, and related complaints. However, studies indicate that awareness of kidney disease remains low in the general population (4). One of the complaints that can increase the burden of this disease and costs in kidney patients is mental disorders (5).

Mental disorders are one of the most common health problems in the world, and the burden caused by them is significant for the health system, societies, and individuals (6). It is estimated that there are 970.1 million cases of mental disorders worldwide (6), which is an increase of 48.1% compared to the past three decades (6). Of these cases, 507.9 million are women and 462.2 million are men (6). Studies on kidney patients have also shown that the prevalence of mental disorders is high in this population (7–9). A national study on a large population of kidney patients has shown that 28.3% have mental disorders (10). A further analysis of the prevalence of depression shows a prevalence of 39.3 and 22.8% based on questionnaires and interviews, respectively (11). One of the most important factors influencing both physical and mental health is lifestyle (12–15).

Lifestyle is a multidimensional, complex, and generic concept that has been defined in various ways in scientific research (16). There is no single definition; however, one definition in line with the current research is “Collective patterns of health-related behavior based on choices from options available to people according to their life chances (17).” A lifestyle includes components such as nutrition, physical activity, smoking, sleep, and stress management (18–20). Any of the unhealthy lifestyle factors can increase the risk of diseases (14). The World Health Organization (WHO) states that 60% of the factors influencing personal health and quality of life are related to lifestyle (21). Unhealthy lifestyle is known to be associated with risk of cancer (22), coronary heart disease (23), stroke (24), ardiometabolic diseases (25), mortality (26), and poor sleep quality (27). On the other hand, a healthy lifestyle is related to improving health-related quality of life (28) and reducing sleep disturbances (29). Inadequate dietary habits and insufficient physical activity stand out as key lifestyle factors linked to adverse health outcomes in individuals living with chronic kidney disease (30). Engaging in physical activity and exercise is highly encouraged for individuals at every stage of chronic kidney disease (31). A recent systematic review and practice guideline highlights that engaging in exercise before dialysis can lead to notable improvements in blood pressure, physical function and capacity, reduction in functional limitations, and an enhanced quality of life related to overall health (32). Considering what has been said, evidence shows the effects of different dimensions of lifestyle on health outcomes in chronic kidney disease patients.

As stated, the mental health status of chronic kidney disease patients is worse compared to the general population. This case–control comparative study examined the mental health status and lifestyle of chronic kidney disease patients in comparison with a healthy control group. Additionally, lifestyle factors were evaluated as risk factors for chronic kidney disease.

## Materials and methods

### Study design and population

This case–control comparative study included chronic kidney disease patients and healthy participants. The CKD patients were recruited from a large hospital, including its kidney ward, while healthy participants were companions of patients who had no disease. This study was conducted face-to-face between June and July 2024.

### Ethical considerations

The research was conducted following ethical standards and in compliance with the guidelines of the Helsinki Declaration (33). This research was approved by the local ethics committee (IR.IAU.SRB.REC.1403.119). All the participants filled out the consent form, and the research executives gave them sufficient explanations about confidentiality. The participants could withdraw from the research at any stage.

### Consent to participate

Informed consent was obtained from all subjects.

### Sample size and calculation

The sample size was calculated using G.power software (34). Based on considering Effect size power 0.50, *α* error probability 0.05, power 0.95, and allocation ratio 1, the required sample size was 88 for each group using G*power software (34). To increase reliability as well as consider possible data loss and missing data, the initial sample size for this research was 100 participants for each group, and information was collected from 200 participants. The research sample was collected using convenience sampling, involving patients with kidney disease and their healthy companions who participated as control subjects.

### Eligibility criteria

The study population (P) consisted of chronic kidney disease patients aged≥18 years and a healthy control group. This research was an observational study. We have a healthy control group as reference (C). Outcomes of interest were health-promoting lifestyle profile, health-related quality of life, psychiatric morbidity, and somatic symptoms experiences.

### Instruments

#### Sociodemographic

A self-report form was used to measure demographic, economic, and social characteristics. This form included age, sex, education, employment status, income status, living with, history of disease, body mass index, smoking, and physical activity.

#### Health-related quality of life

The Short-Form 36 (SF-36) is used to measure health-related quality of life. This questionnaire includes 36 items, with total scores between 0 and 100; higher scores indicate a higher health-related quality of life. This questionnaire consists of eight subscales: physical functioning, role limitations due to physical health, role limitations due to emotional problems, energy and fatigue, mental health, social functioning, pain, and general health (35–37). Cronbach’s alpha coefficients range from 0.77 to 0.90, except for the vitality scale (alpha = 0.65) (38).

#### Health-promoting lifestyle profile

The health-promoting lifestyle questionnaire has 52 items, which are grouped into six subscales: “health responsibility (nine items), spiritual growth (nine items), physical activity (eight items), interpersonal relationships (nine items), nutrition (nine items), and stress management (eight items)” (39). This questionnaire was developed in 1987 by Walker et al. (39), and its psychometric properties have been evaluated. The scoring of this questionnaire is based on a 4-point Likert scale: never (1), sometimes (2), frequently (3), and regularly (4) (39), with higher scores indicating healthier lifestyle behaviors (39). Cronbach’s alpha of 0.94 was reported for this questionnaire, and the subscale of Cronbach’s alpha was between 0.79 and 0.87 (39). The lifestyle variable is a variable with an ordinal scale. A score between 104 and 152 is classified as a poor lifestyle, 105 and 156 as a moderate lifestyle, and 157 and 208 as a good lifestyle (40).

#### General Health Questionnaire

The General Health Questionnaire (GHQ) is one of the most common questionnaires for measuring psychiatric morbidity (41). This questionnaire contains 28 items that are scored based on a four-point Likert scale (0-not at all, 1-no more than usual, 2-rather more than usual, and 3-much more than usual). The four subscales of this questionnaire include somatic symptoms, anxiety/insomnia, social dysfunction, and severe depression (41). High scores in this questionnaire indicate psychiatric morbidity (41). This questionnaire has been used in different cultures and populations, and the findings show its reliability and validity (42). Test–retest reliability has been reported to be high (0.78–0 0.9) and Cronbach’s *α* ranges from 0.9 to 0.95 (43, 44).

#### Somatic Symptoms Experiences Questionnaire

The Somatic Symptoms Experiences Questionnaire (SSEQ) was developed to measure the psychological processes of the somatoform disorder (45). This questionnaire includes 13 items, with four subscales: health worries (2,4,7,9,10), experience of illness (1,5), problems while interacting with physicians (3,6,8), and consequences of illness (11,12,13). The Likert scoring of this questionnaire is six degrees: never, very rarely, rarely, often, very often, and always. Higher scores indicate greater experience of somatic symptoms (45). Cronbach’s *α* = 0.90 was reported for this questionnaire (45). In other cultures, this questionnaire has good validity and reliability (46).

### Statistical analysis

We conducted two-part analyses for further insight. The first part was a comparison between chronic kidney disease patients and the healthy control group based on variables, and the second part was an examination of risk factors for chronic kidney disease. In this research, the exposure variable included health promotion lifestyle, and the outcome was health-related quality of life, mental health, and somatic symptoms experiences. At first, an analysis of the sociodemographic status of the participants was performed. The association between sociodemographic and health behaviors with the risk of kidney disease was investigated using logistic regression, and odds ratios with 95% confidence intervals were calculated. An independent *t*-test was used to compare chronic kidney disease patients and the healthy control group in terms of lifestyle, health-related quality of life, mental health, and somatic symptoms experiences. The zero-order correlation coefficient and linear regression were used to examine the association between health promotion lifestyle with health-related quality of life, mental health, and somatic symptoms experiences. SPSS 27 (IBM, United States) and Stata-14 software (Stata Corp. College Station, TX) were used for software statistical calculations.

## Results

Table 1 shows the demographic and health status of individuals with chronic kidney disease and the healthy control group. The majority of participants in this study were male, and the prevalence of overweight was very high, particularly in terms of body mass index. The majority of the participants reported a history of disease, and physical activity was low among the participants.

**Table 1:** Demographic and health status of the participants stratified by groups.

Variable	Chronic kidney disease	Healthy control group
Gender		
Men	56%	93%
Women	44%	7%
Age		
18–24 years	3%	29%
24.1–40 years	26%	59%
40 and higher years	71%	12%
Body mass index		
Underweight	3%	1%
Normal weight	48%	43%
Overweight	42%	49%
Obesity	7%	7%
Education		
Illiterate	28%	1%
Diploma and less	55%	45%
Bachelor’s degree	7%	45%
Master’s or Ph.D	8%	4%
Unknown	2%	5%
Employment status		
Employment	20%	77%
Retired	27%	2%
Unemployment	51%	19%
Unknown	2%	2%
Income status		
Excellent	7%	–
Good	13%	50%
Moderate	48%	42%
Poor	32%	8%
Live with		
Alone	8%	8%
With family	89%	92%
Unknown	3%	–
History of disease		
Yes	70%	43%
No	29%	56%
Unknown	1%	1%
Smoking status		
Former	22%	14%
Current	9%	37%
Never	69%	48%
Unknown	–	1%
Physical activity class		
Low	71%	75%
Moderate	23%	20%
High	6%	5%

According to the results shown in Table 2, chronic kidney disease patients had lower scores in most aspects of lifestyle and health-related quality of life compared to the healthy control group, which means that health-related quality of life is low and the lifestyle is worse. Moreover, the chronic kidney disease group showed a worse condition in terms of mental health than the healthy control group.

**Table 2:** Means and standard deviations of health-related quality of life, lifestyle, and mental health variables in chronic kidney disease and healthy control groups.

Variables	Group	Mean	SD	Standard error of mean
Physical functioning	Chronic kidney disease	45.2954	30.81025	3.08103
	Normal	78.8735	22.52505	2.25251
Role physical functioning	Chronic kidney disease	39.1097	38.81438	3.88144
	Normal	62.6301	41.94344	4.19434
Role emotional functioning	Chronic kidney disease	42.2527	41.72980	4.17298
	Normal	64.0342	41.82718	4.18272
Vitality	Chronic kidney disease	49.3890	24.45766	2.44577
	Normal	56.3229	16.48399	1.64840
Mental health	Chronic kidney disease	59.6512	23.97099	2.39710
	Normal	59.4000	14.97068	1.49707
Social functioning	Chronic kidney disease	54.7914	29.44716	2.94472
	Normal	68.0414	20.58401	2.05840
Bodily pain	Chronic kidney disease	50.2750	31.27125	3.12712
	Normal	71.4000	24.10866	2.41087
General health	Chronic kidney disease	38.5237	17.66321	1.76632
	Normal	56.2500	14.18448	1.41845
Spiritual growth	Chronic kidney disease	1.6009	0.77413	0.07741
	Normal	2.2350	0.84825	0.08482
Health responsibility	Chronic kidney disease	1.6287	0.70762	0.07076
	Normal	1.9665	0.64292	0.06429
Physical activity	Chronic kidney disease	1.3192	0.59859	0.05986
	Normal	1.4955	0.55871	0.05587
Nutrition	Chronic kidney disease	2.0463	0.65185	0.06519
	Normal	1.8894	0.53199	0.05320
Interpersonal relations	Chronic kidney disease	1.8002	0.83737	0.08374
	Normal	2.1788	0.65482	0.06548
Stress management	Chronic kidney disease	1.7367	0.58969	0.05897
	Normal	1.8064	0.55317	0.05532
Health worries	Chronic kidney disease	14.1409	5.48049	0.54805
	Normal	6.7901	4.50015	0.45002
Experience of illness	Chronic kidney disease	6.1262	2.56728	0.25673
	Normal	2.6815	2.06921	0.20692
Problems while interacting with physicians	Chronic kidney disease	3.8091	3.49234	0.34923
	Normal	2.0600	2.27778	0.22778
Consequences of illness	Chronic kidney disease	8.8506	4.12548	0.41255
	Normal	4.3200	2.73356	0.27336
Somatic symptoms	Chronic kidney disease	1.2729	0.65465	0.06546
	Normal	0.8855	0.57819	0.05782
Anxiety and sleep disorder	Chronic kidney disease	1.1587	0.76546	0.07655
	Normal	0.6373	0.60440	0.06044
Social dysfunction	Chronic kidney disease	1.4657	0.68500	0.06850
	Normal	1.3661	0.44749	0.04475
Severe depression	Chronic kidney disease	0.8130	0.97009	0.09701
	Normal	0.5900	0.78867	0.07887

In Table 3, the health-related quality of life, lifestyle, somatic symptoms experiences, and mental health have been compared between chronic kidney disease patients and the healthy control group, and the results showed that chronic kidney disease patients had worse conditions in health-related quality of life and lifestyle than the healthy control group. Moreover, chronic kidney disease patients have more somatic symptoms experiences and mental disorders than the healthy control group.

**Table 3:** Comparison of health-related quality of life, lifestyle, and mental health in chronic kidney disease patients and healthy control group.

Variables		t	Significant	Mean difference	95% confidence interval of the difference		Hedges’ g
					Lower	Upper	
Health-related quality of life	Physical functioning	−8.798	0.000	−33.57807	−41.10875	−26.04739	1.24
	Role physical functioning	−4.116	0.000	−23.52040	−34.79035	−12.25044	0.58
	Role emotional functioning	−3.687	0.000	−21.78156	−33.43298	−10.13014	0.52
	Vitality	−2.351	0.020	−6.93384	−12.75517	−1.11252	0.33
	Mental health	0.089	0.929	0.25120	−5.32869	5.83108	0.01
	Social functioning	−3.688	0.000	−13.25000	−20.34025	−6.15975	0.52
	Bodily pain	−5.350	0.000	−21.12500	−28.91474	−13.33526	0.75
	General health	−7.825	0.000	−17.72632	−22.19494	−13.25770	1.10
Lifestyle	Spiritual growth	−5.521	0.000	−0.63405	−0.86053	−0.40758	0.78
	Health responsibility	−3.533	0.001	−0.33776	−0.52631	−0.14921	0.49
	Physical activity level	−2.153	0.033	−0.17629	−0.33777	−0.01482	0.30
	Nutrition	1.865	0.064	0.15693	−0.00903	0.32289	0.26
	Interpersonal relations	−3.561	0.000	−0.37855	−0.58825	−0.16885	0.50
	Stress management	−0.861	0.390	−0.06963	−0.22908	0.08982	0.12
Somatic symptoms experiences	Health worries	10.366	0.000	7.35079	5.95203	8.74954	1.46
	Experience of illness	10.447	0.000	3.44462	2.79420	4.09505	1.47
	Problems while interacting with physicians	4.195	0.000	1.74912	0.92607	2.57218	0.59
	Consequences of illness	9.155	0.000	4.53063	3.55377	5.50748	1.29
Mental health	Somatic symptoms	4.436	0.000	0.38742	0.21517	0.55968	0.62
	Anxiety and sleep disorder	5.346	0.000	0.52141	0.32901	0.71380	0.75
	Social dysfunction	1.218	0.225	0.09967	−0.06184	0.26119	0.17
	Severe depression	1.784	0.076	0.22302	−0.02359	0.46963	0.25

**Figure 1:**
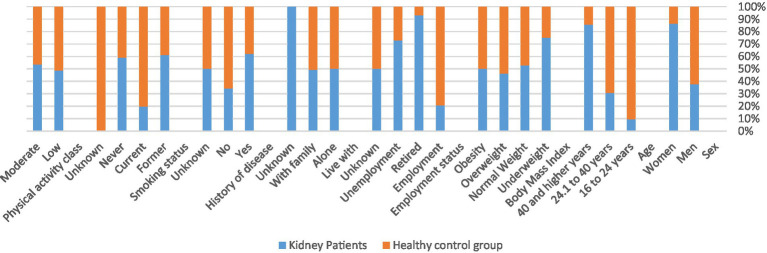
Distribution of characters in kidney and healthy population.

The health status of chronic kidney disease patients compared to the healthy control group is shown in Table 4. Factors associated with kidney disease risk include female gender, history of disease, and being retired (Figure 1).

**Table 4:** Odds ratio of kidney disease based on associated variables.

Group	Odds ratio	Std. Err.	z	P > z	[95% CI]	
Gender						
Men	Ref					
Women	10.43	4.60	5.32	0.001	4.40	24.76
Age						
24–40 years	Ref					
16–24 years	0.23	0.15	−2.23	0.026	0.06	0.84
≥40 years	13.42	5.24	6.64	0.001	6.24	28.88
History of disease						
No	Ref					
Yes	3.09	0.91	3.80	0.001	1.72	5.53
Body mass index						
Normal weight	ref					
Underweight	2.68	3.15	0.84	0.400	0.26	26.81
Overweight	0.76	0.22	−0.89	0.374	0.42	1.37
Obesity	0.89	0.51	−0.19	0.848	0.29	2.76
Smoking						
Never	Ref					
Current	1.09	0.42	0.23	0.819	0.50	2.34
Former	0.16	0.068	−4.34	0.001	0.07	0.37
Live with						
Family	Ref					
Alone	1.00	0.52	0.00	1.000	0.35	2.77
Physical activity						
Moderate	Ref					
Low	0.82	0.28	−0.56	0.576	0.41	1.62
High	1.04	0.70	0.06	0.950	0.27	3.94
Employment						
Unemployment	Ref					
Employment	0.10	0.03	−6.28	0.001	0.05	0.21
Retired	5.02	3.92	2.07	0.039	1.08	23.22

Prediction of health-related quality of life, mental health, and somatic symptoms based on lifestyle showed that health-promoting lifestyle in chronic kidney disease patients had a direct relationship with high health-related quality of life. Moreover, a health-promoting lifestyle was negatively related to mental disorders and somatic symptoms experiences (Table 5).

**Table 5:** Prediction of health-related quality of life, somatic symptoms, and mental health by lifestyle profile in chronic kidney disease patients.

Variables	Coefficient	Unstandardized coefficients		Standardized coefficients	t	Sig.	95.0% CI for B		R^2^
	Zero-order	B	Std. Error	Beta			Lower bound	Upper bound	
Mental component									
Lifestyle profile	0.50**	0.36	0.06	0.50	5.78	0.001	0.24	0.49	0.25
Physical component									
Lifestyle profile	0.60**	0.42	0.06	0.60	7.45	0.001	0.31	0.54	0.36
Mental health									
Lifestyle profile	−0.51**	−0.10	0.002	−0.51	−5.86	0.001	−0.014	−0.007	0.26
Somatic symptoms									
Lifestyle profile	−0.43**	−0.15	0.03	−0.43		0.001	−0.218	−0.091	0.19

**p < 0.01.

## Discussion

This research was conducted to investigate the lifestyle and mental health of chronic kidney disease patients, as well as the relationship between lifestyle and the risk of mental health problems in these patients. A total of 100 chronic kidney disease patients and 100 healthy individuals participated in this study. The examination of health-related quality of life in both groups showed that, except for the mental health component, chronic kidney disease patients had a lower health-related quality of life than the healthy control group in all other components. Health-related quality of life is one of the characteristics that have been considered for measuring the effectiveness of treatment on patients (47). The findings obtained from this research are consistent with the study that examined the health-related quality of life in different stages of chronic kidney disease (47, 48). The results of this study pertain to patients who currently have chronic kidney disease.

Another finding obtained from this research showed an unhealthy lifestyle in kidney patients compared to the healthy control group, and this difference was significant. This finding aligns with previous studies that also report an unhealthy lifestyle in kidney patients compared to healthy controls (49). There is a relationship between unhealthy lifestyles and non-communicable diseases, and it has been hypothesized that a similar mechanism is responsible for the relationship between unhealthy lifestyles and kidney disease (49). The connection between unhealthy lifestyle behaviors and chronic kidney disease appears to stem from shared underlying mechanisms, where the cumulative impact of these behaviors likely contributes significantly to the development of the condition. However, given the cross-sectional design of the current study, establishing a definitive causal relationship between unhealthy lifestyle habits and kidney disease prevalence was not feasible (49). For example, a meta-analysis has shown that reduced physical activity as a lifestyle component affects the development of kidney disease through obesity, hypertension, and type 2 diabetes (50). A similar finding was observed for other unhealthy lifestyle components, including poor nutrition, characterized by high levels of animal fat, sodium, and soft drinks, all of which cause the development of renal dysfunction (51–53). In this regard, studies have shown that in kidney patients, lifestyle modifications, including regular physical activity, weight loss, and smoking cessation, are associated with a lower risk of adverse outcomes (32, 54–56).

Mental health problems are a significant concern among chronic kidney disease patients (7), and based on this, the present study investigated mental disorders in these patients. The chronic kidney disease patients in this study reported more pain, physical complaints, and depression than the healthy control group, although the differences in depression were not significant. These findings about mental health issues in chronic kidney disease patients are consistent with previous studies (10, 57, 58). Mechanisms on the effects of kidney disease and its stages on mental disorders have been described (7). For example, an increase in inflammatory molecules, reactive oxygen species, and angiotensin II can affect brain cells and thus affect mental disorders (5). In another part of the findings obtained from this study, it has been shown that a healthy lifestyle is associated with an increase in the health-related quality of life in kidney patients. In this regard, studies have also shown that a healthy lifestyle is a factor that increases the health-related quality of life (59). In this context, meta-analysis studies have also shown that lifestyle interventions are associated with an increase in health-related quality of life (28). Furthermore, a healthy lifestyle has been associated with an increase in mental health and wellbeing (13, 60). A healthy lifestyle can be effective in the prevention and treatment of many physical and mental health conditions, and compared to other treatments used for mental disorders, such as drug therapy or psychological therapy, it can have long-lasting effects and impose a lower cost burden on the individual. Therefore, it is necessary to pay more attention to lifestyle and its components, especially considering the changes that have taken place in various social and occupational dimensions over recent decades.

## Limitations

This case–control comparative study investigated the dimensions of health-related quality of life and mental health in chronic kidney disease patients compared to the healthy control group. Due to the nature of this research, which was cross-sectional, this issue should be kept in mind in generalizing the results, and also in future studies, longitudinal studies and interventions based on lifestyle can be insightful. This study was conducted at a single point in time, and at the time of the study, the majority of the patients in the renal unit were women, which is a notable limitation. Therefore, gender may have influenced the findings, and this should be considered when interpreting the results. To investigate the risk factors for CKD, the best design is a longitudinal cohort study.

## Data Availability

The raw data supporting the conclusions of this article will be made available by the authors, without undue reservation.
